# Entrepreneurial motivations and business performance: A study of female online microbusiness owners

**DOI:** 10.1371/journal.pone.0289946

**Published:** 2023-08-11

**Authors:** Shibo Li, Edwin Setiawan Sanusi

**Affiliations:** 1 Department of Management, SolBridge International School of Business, Daejeon, Republic of Korea; 2 Department of Management and Economics, Liaoning University of Technology, Jinzhou, Liaoning Province, China; COMSATS University Islamabad - Wah Campus, PAKISTAN

## Abstract

This study aims to examine the correlation between various types of entrepreneurial motivations and the corporate performance of self-employed micro-businesses operated by women in China. Through the application of Structural Equation Modeling (SEM) estimation on a sample of 160 female entrepreneurs, our findings reveal that female entrepreneurs driven by pull motivation prioritize non-financial performance as their primary goal. Conversely, those driven by push motivation exhibit a greater emphasis on financial performance. Furthermore, the cross-group analysis indicates that a high level of motivation among necessity-driven female microbusiness entrepreneurs contributes to achieving a high level of financial performance, whereas a high level of motivation among opportunity-based female microbusiness entrepreneurs does not significantly influence non-financial performance. The implications of these findings for research and policy development pertaining to Chinese female online microbusinesses are also discussed.

## Introduction

According to Fu [1], Chinese women are increasingly turning to microbusiness entrepreneurship as a means of overcoming workplace discrimination and achieving a better work-life balance. The definition of microbusiness has evolved with the changing landscape of social media on the internet. Presently, microbusinesses are defined as enterprise merchants who conduct mobile e-commerce through social media platforms such as WeChat, Weibo, among others [2]. In China, this field employs over 60 million people, with more than 50% being women, and the transaction volume exceeded $2 trillion in 2019. Additionally, research conducted by Minniti [3] and Zhang [4] shows that the majority of self-employed online microbusiness owners in China are female.

Despite the significant increase in female microbusinesses in China, there is still a dearth of research on this topic. Several studies have pointed out the scarcity of research related to female entrepreneurship, particularly in the context of China [5–7]. Moreover, despite the growing interest in female entrepreneurship, there is still a significant lack of research on the impact of different types of motivation on various types of business performance, particularly in the context of female entrepreneurship [8–10]. While there has been some research exploring the relationship between motivation and business performance [11], little is known about how different types of entrepreneurial motivation influence specific aspects of business performance [12].

To address these gaps, this study aims to investigate the relationship between different types of entrepreneurial motivation and the performance of female self-employed microbusinesses in China. Specifically, the study will focus on two types of motivation: necessity-driven entrepreneurship and opportunity-based entrepreneurship [13,14]. Necessity-driven entrepreneurship refers to individuals being pushed into entrepreneurship due to limited employment options or financial constraints, while opportunity-based entrepreneurship refers to individuals seeking entrepreneurial opportunities driven by factors such as autonomy, passion, and self-development [13,14].

The study will examine the impact of these two types of motivation on two dimensions of business performance: financial performance and non-financial performance. Financial performance refers to the financial outcomes of the business, such as profitability and revenue growth [15,16]. Non-financial performance, on the other hand, encompasses aspects such as innovation, customer satisfaction, and employee engagement [15,16].

Additionally, the study will explore the mediating effect of non-financial performance on the relationship between motivation and financial performance. It is hypothesized that non-financial performance acts as a mediator, meaning that the positive impact of motivation on financial performance is partially explained by the influence of non-financial performance [15,16].

This research is important and worth investigating as it will contribute to the understanding of the interplay between different types of motivation and business performance in the context of female entrepreneurship in China. By examining the specific impacts of necessity-driven and opportunity-based entrepreneurship on financial and non-financial performance, this study can provide valuable insights for policymakers, practitioners, and aspiring entrepreneurs. The findings can inform the development of targeted strategies and interventions to support the growth and success of female self-employed microbusinesses in China. Furthermore, this research will contribute to the existing literature on entrepreneurial motivation and performance by examining the specific impacts of different types of motivation on various aspects of business performance.

In conclusion, this study aims to investigate the relationship between different types of entrepreneurial motivation and the performance of female self-employed microbusinesses in China. The research objectives include examining the impact of necessity-driven and opportunity-based entrepreneurship on financial and non-financial performance, as well as exploring the mediating effect of non-financial performance on the relationship between motivation and financial performance. The study’s findings will provide valuable insights for policymakers, practitioners, and aspiring entrepreneurs, and contribute to the existing literature on entrepreneurial motivation and performance.

In the following parts of this manuscript, we will perform a thorough literature review that has led to the development of our hypotheses. Afterward, we will proceed to validate our hypotheses by analyzing survey data and explaining the main findings. Finally, this study explores its contributions, acknowledges limitations, and suggests directions for future research.

## Literature review

### Female entrepreneurial motivation

Women’s entrepreneurial motivation is similar to that of their male counterparts, and is defined as the process that initiates, guides, and maintains goal-oriented behaviors [17]. Both male and female entrepreneurs can be motivated by either necessity or opportunity. Push factors, such as unemployment or job loss, can prompt individuals to start their own businesses, often referred to as "necessity" entrepreneurs. On the other hand, pull factors such as recognizing a business opportunity or being inspired by a mentor or role model can propel individuals to venture into entrepreneurship. These entrepreneurs seize emerging opportunities and are commonly known as "opportunity" entrepreneurs [18,19]. This system of taxonomy has found widespread use, with the Global Entrepreneurship Monitor (GEM) using this classification since 2001 [20].

Although women and men are driven by similar factors, the reasons why women opt for entrepreneurship are somewhat distinct. According to Arai [21] and Mallon and Cohen [22], women are expected to fulfill both family provider and child-rearing roles. Additionally, workplace discrimination often forces women to leave paid employment due to the presence of the glass ceiling, which impedes their advancement to top management positions [23,24]. Researchers suggest that push factors for women in entrepreneurship include dissatisfaction with current employment (including the effects of the glass ceiling), flexibility, family responsibilities, and financial necessity (such as being a single parent or having an unemployed husband) [25]. In contrast, pull factors represent a different form of entrepreneurial motivation where women voluntarily choose entrepreneurship, sometimes leaving behind well-paying jobs. Hankinson [25] propose that pull factors for women in entrepreneurship include independence, self-fulfillment, and income pursuit. While women are driven by both push and pull motivations to engage in entrepreneurship, women have a higher likelihood of being pushed into entrepreneurship, as reported by some researchers due to factors like lower educational attainment, fewer job experiences, or more frequent career interruptions [26].

The available evidence suggests that female entrepreneurs are more likely to be motivated by "push" factors based on necessity compared to their male counterparts. Studies conducted in the United States have found that female entrepreneurs are typically less driven than male entrepreneurs to control their own destinies, be their own bosses, make income, or have power [27]. Instead, female entrepreneurs tend to view their businesses as a way of achieving work-life balance [28]. Similar findings have been reported in other developed nations, where women entrepreneurs are more likely to be driven by necessity [29]. According to the most recent GEM data analyzed by Kelley et al. [26], globally, women are more likely than men to be motivated by necessity, with this trend being especially prominent in less developed economies. In less developed nations, female entrepreneurs often start their own businesses for survival, nutrition, healthcare, or family-related educational reasons, while in developed economies, they are dissatisfied with the lack of career growth opportunities [30,31]. The factors driving entrepreneurship appear to differ depending on a country’s level of economic development. Female entrepreneurs in less developed economies are more likely to be driven by necessity, while those in more developed economies are more likely to pursue opportunities [32].

### Goal-setting theory

Goal-setting theory, initially proposed by Edwin A. Locke and Gary P. Latham in 1990 [33], is a well-established framework that explores the relationship between goals, motivation, and performance. The theory highlights the intrapersonal nature of goal setting and its impact on human behavior and achievement. According to the general proposition of the theory, goals originate from personal values, which then evoke emotions and desires within individuals [34]. These emotions and desires subsequently motivate individuals to establish specific goals or intentions [34]. The theory distinguishes between the intrapersonal and interpersonal aspects of goal setting. The intrapersonal nature of goal setting refers to the process by which individuals internally establish goals based on their personal values, desires, and aspirations [34]. On the other hand, the interpersonal nature of goal setting involves the external influence and social context that shape an individual’s goals, such as feedback and expectations from others [35].

Goal-setting theory proposes four mechanisms through which goals influence motivation and performance. Firstly, goals direct attention by guiding individuals to prioritize activities that are relevant to their goals. This selective attention ensures that individuals focus their efforts on tasks that align with their objectives [36]. Secondly, goals mobilize energy by creating a sense of purpose and arousal, motivating individuals to channel their efforts effectively towards goal achievement [34]. Thirdly, goals enhance persistence by fostering commitment and determination. The clarity and specificity of goals provide individuals with a benchmark for progress, enabling them to persevere through obstacles and setbacks [36]. Lastly, goals lead to the adoption of behavior and strategies that are conducive to goal accomplishment. Individuals develop and implement action plans, strategies, and task-specific behaviors to achieve their goals [37].

A new development in the goal-setting theory was the inclusion of primed or subconscious goals. Previously, it was believed that only intentionally set goals had motivational impact. Building on Bargh and Chartrand’s automatic theory [38,39] and McClelland’s implicit motives theory [40], Locke and Latham recognized that subconscious goals could be triggered without conscious control over their effects on behavior [41]. Numerous studies have supported the efficacy of these primed goals. For instance, Itzchakov and Latham [42] conducted a study examining the relationship between subconscious goal priming and performance through three laboratory experiments and one field experiment. They discovered that subconscious goals not only form the foundation for the difficulty level of consciously set goals but also interact with conscious goals to influence performance. Another relevant study by Lowery et al. [43] conducted two experiments where participants were subliminally primed with words related or unrelated to intelligence before taking a practice exam, administered 1 to 4 days before an actual course midterm. The results demonstrated that intelligence primes enhanced performance on the midterm compared to neutral primes. Collectively, these studies confirm the significance of incorporating subconscious goals into the goal-setting theory as a motivating factor that impacts performance.

### Business performance in female microbusinesses

The evaluation of business performance is predominantly based on financial metrics such as profits, revenues, return on investment, return on sales, and return on equity [15]. Objective measurements are used to assess organizational performance, which is primarily focused on financial gains. Although financial metrics provide a clear and tangible way to measure performance, Dess and Robinson [44] argue that most empirical research on the relationship between strategic management techniques and organizational performance has confused "performance" with "success." While success is a crucial goal for businesses, it is just one aspect of performance, and other aspects such as customer satisfaction and employee engagement should also be considered. In any case, financial measures remain a vital tool for measuring business performance and are commonly used to assess metrics like profit, revenue, cash flow, return on equity, and growth [15].

Research indicates that women-owned enterprises tend to have lower profitability and slower growth compared to their male counterparts [27,45]. However, the decision to grow a business is not solely based on financial success. Morris et al. [46] and Costin [47] suggest that many female business owners prioritize objectives other than growth, and they may choose not to expand their enterprises. Growth can encompass more than just increasing the size of a business. It can also mean personal and professional development, such as growing in knowledge and ability. Therefore, when evaluating the performance of women-owned businesses, financial indicators should not be the only factor considered. Evaluators should also consider non-financial indicators like employee satisfaction, social contributions, goal achievement, and overall effectiveness.

Another contributing factor to the relatively lower performance of female business owners compared to their male counterparts is their inclination to operate within business sectors that offer limited growth opportunities. Research indicates that women-owned businesses often concentrate in labor-intensive sectors such as trade and services, which may have lower potential for growth and development compared to capital-intensive manufacturing industries [48]. This concentration within specific sectors can be attributed to barriers women face in accessing finance, as well as their comparatively lower physical and reputational collateral, which restricts their funding opportunities [48]. Furthermore, women entrepreneurs may encounter challenges related to financial capital, education, work experience, family responsibilities, and gender-related issues, all of which can impact their performance [49].

## Hypotheses development

### Female entrepreneurial motivation and business performance

Building on the goal-setting theory, our proposition suggests that entrepreneurs driven by push motivation, arising from financial pressure, job loss, or the responsibility to support their families, are more prone to engaging in behaviors that contribute to financial success. Such behaviors encompass diligent work, calculated risk-taking, and actively pursuing growth opportunities [48]. The goal-setting theory operates through four different mechanisms, wherein heightened focus and attention lead to the development of strategies aimed at achieving the goals [34,36,37]. These strategies, in turn, generate increased effort directed towards the goals, resulting in prolonged and persistent endeavors. Consequently, this heightened effort and focus lead to enhanced financial performance in their businesses [37].

Previous research strongly supports the idea that goal setting enhances effort and attention, thereby leading to high performance. One notable meta-analysis conducted by Tubbs [49] examined eighty-seven studies, comprising both laboratory experiments and field survey studies. The findings revealed that goal specificity, goal difficulty, participative goal setting, and feedback all positively correlated with performance. Similarly, Epton and colleagues [50] conducted another meta-study, analyzing 141 papers that involved randomized laboratory trials. The studies measured behavior change in various domains, such as cognitive, sporting, and production goals. The study found that goal setting consistently exhibited statistically significant effects on behavioral change. Furthermore, McEwan and colleagues [51] conducted their meta-analysis, encompassing 45 articles that used randomized laboratory trials. The analysis demonstrated an overall medium positive effect of goal setting on physical activity behavior.

The second argument supporting this relationship corresponds with the latest development in the goal-setting theory, which introduces primed goals as an additional motivational factor alongside intentional and conscious goals [52]. Goals related to business survival and securing a primary income source can have an impact without being intentionally established. According to the influence of primed goals, as supported by the automaticity theory [38,39] and the implicit motives theory [40], entrepreneurs who embark on business ventures out of necessity have this information stored in their memory. Consequently, these subconscious memories influence their efforts and strategies, with a primary focus on financial objectives.

Past studies have provided support for the effectiveness of these primed goals. For example, Itzchakov and Latham [42] conducted a study investigating the link between subconscious goal priming and performance through three laboratory experiments and one field experiment. They found that subconscious goals not only lay the groundwork for the difficulty level of consciously set goals but also interact with conscious goals to influence performance. Another relevant study by Lowery et al. [43] carried out two experiments where participants were subliminally primed before taking an exam. The results revealed that the primes enhanced performance on the midterm compared to neutral primes. Lastly, a study by Shantz and Latham [53,54] focused on employees in a call center who were soliciting money for a university. Using a 2 × 2 factorial design, employees were assigned specific monetary goals to attain. The experimental group, which received printed directions with a backdrop photograph of a woman winning a race, showed a significant increase in performance at the end of the work shift. Altogether, these studies confirm the importance of subconscious goals as a motivating factor that significantly impacts performance.

On the flip side, entrepreneurs driven by pull motivation, which arises from a desire for autonomy, creativity, or social impact, tend to engage in behaviors that lead to non-financial success [34]. These behaviors encompass the development of innovative products, the establishment of strong customer relationships, and contributions to their communities. Moreover, individuals who embark on entrepreneurial endeavors driven by opportunities aim to fulfill social, esteem, and self-fulfillment needs [48,55]. This heightened focus on non-financial outcomes can lead to elevated levels of non-financial performance in their businesses [34,36]. Similarly, in line with the impact of primed goals as supported by the automaticity theory [38,39] and the implicit motives theory [40], entrepreneurs who initiate businesses out of passion and the aspiration for independence retain these factors in their memory. Consequently, these subconscious memories influence the efforts and strategies of entrepreneurs, primarily directed towards goals related to non-financial aspects [52]. Such goals may include facilitating employment opportunities for others or personal growth, such as acquiring knowledge about business and leadership.

The significance of an entrepreneur’s motivation on business performance is also supported by previous research in the field of entrepreneurship. Zhao et al. [56] confirmed the notion that entrepreneurial motivation, measured as entrepreneurial intention, varies across individuals. Their study, which involved a sample of master of business administration students from 5 universities, found that entrepreneurial motivation differs due to variations in perceived entrepreneurial learning, previous entrepreneurial experience, risk propensity, and gender. All four antecedents were found to have a significant effect on entrepreneurial intention through the mediating effect of self-efficacy, except for gender, which directly influenced intention. This study provides evidence that entrepreneurial motivation varies among individuals across different dimensions.

Collins et al. [57] conducted a meta-analysis to examine their main proposition that achievement motivation has a significant positive influence on both the choice of an entrepreneurial career and entrepreneurial performance. They argued that individuals with a high need for achievement are more likely to engage in instrumental activities relevant to high entrepreneurial performance. The study found that both career choice as an entrepreneur and entrepreneurial performance are significantly affected by the need for achievement. This study is important as it supports our hypothesis that people’s motivation is related to entrepreneurial performance because needs and values drive effort, resulting in performance.

Similarly, Machmud and Sidharta [11] examined the relationship between the need for achievement and business performance of SMEs. They proposed that individuals high in the need for achievement are more likely to engage in tasks with a high probability of leading to high performance. Their hypothesis that achievement motivation is related to entrepreneurial performance was validated in the study. This study is important for our argument as it provides evidence that motivation is indeed related to entrepreneurial performance. Building on the literature review, we advance the following hypotheses:

Hypothesis 1 (H1): Entrepreneurial push motivation positively influences business financial performance.

Hypothesis 2 (H2): Entrepreneurial pull motivation positively influences business non-financial performance.

### The central mechanism of non-financial performance

According to the Resource Based View (RBV) [58,59], strong financial performance, even in small- and medium-sized enterprises [60], relies on investments in valuable, rare, non-imitable, and non-substitutable resources, such as human capital, new technologies, and new product development. This theory emphasizes the significance of non-financial performance in SMEs to achieve financial success. Non-financial measures like customer satisfaction, employee engagement, and product quality serve as early indicators of the potential long-term success of strategic initiatives [61]. By monitoring and enhancing non-financial performance, companies can increase their likelihood of achieving financial objectives, making it a precursor to long-term financial success.

Furthermore, various studies, including those by Ittner and Larcker [62], Kaplan and Norton [16], and Pham [63], among others, have demonstrated the mediating role of non-financial performance in the impact of strategic initiatives on financial performance. Ittner and Larcker [62] conducted a study on customer, business, and firm levels, revealing positive relationships between customer satisfaction measures and future accounting performance, such as revenue and market value. Kaplan and Norton [16] reported how companies that developed a balanced scorecard, a strategic map linking non-financial to financial performance, experienced improved bottom lines. Pham [63] analyzed 211 companies in Vietnam, focusing on the relationships between Total Quality Management (TQM) practices and financial and non-financial performance. The study found that non-financial performance, measured by improved quality, innovativeness, and market share, fully mediated the relationship between TQM practices and financial performance. Notably, around 50 percent of the sample consisted of small- and medium-sized companies (SMEs). Thus, we propose:

Hypothesis 3a (H3a): Business non-financial performance has a positive influence on business financial performance.

Hypothesis 3b (H3b): Business non-financial performance significantly mediates the relationship between entrepreneurial pull motivation and business financial performance.

According to Irene [64], "push" entrepreneurs often embark on their entrepreneurial journeys unprepared for the challenges of running a business and with limited knowledge of the dynamics and operations of the business environment. In contrast, those who are "pulled" into entrepreneurship typically have better preparation and awareness of the business environment [64]. This suggests that female business owners who seize opportunities are likely to possess more management expertise. The human capital theory [65] aligns with this notion, suggesting that individuals with more knowledge possess superior cognitive abilities, making them more productive and efficient workers. However, for female business owners, growth is often a conscious decision, as stated by Morris [46], with many choosing to prioritize other objectives over expanding their enterprises. As a result, female "pulled" entrepreneurs, despite possessing the human capital to excel financially, may not always achieve significant financial success. Nevertheless, they have the potential to succeed financially. Therefore, it is proposed that entrepreneurial pull motivation positively influences business financial performance.

Hypothesis 4 (H4): The entrepreneurial pull motivation has a positive influence on business financial performance.

**Fig 1:**
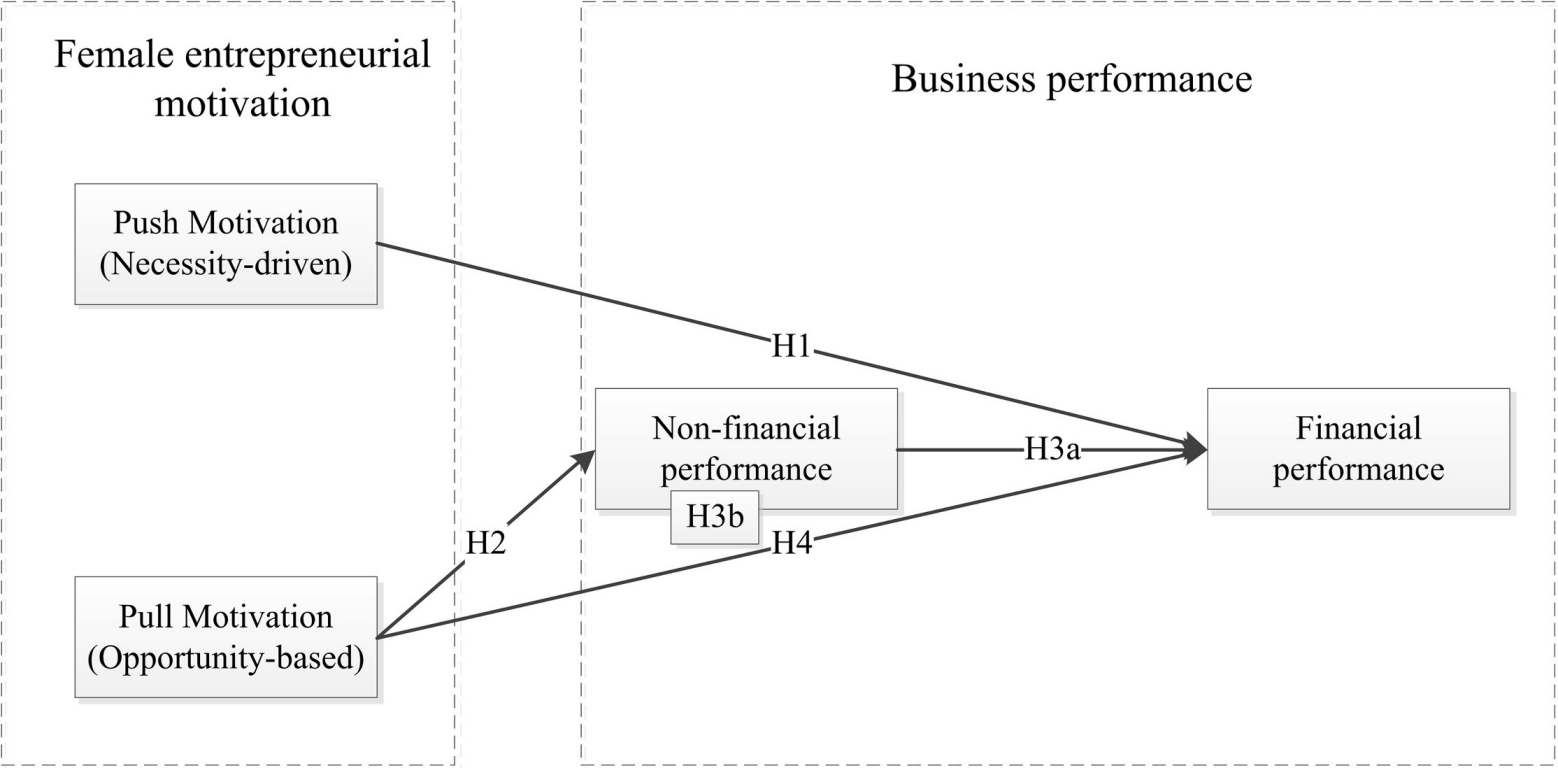
Theoretical model.

In sum, we propose a model in which business non-financial performance plays a critical mediating role (Fig 1).

## Methodology

### Sample and data collection

This study focuses on female entrepreneurs in online microbusiness self-employment in Liaoning Province, China. The preliminary population consists of women surveyed mainly from Liaoning’s Union of Beauty, a non-governmental women’s organization that aims to organize community activities for women’s public welfare. These activities include etiquette classes, health lectures, cultural performances, and product matching, among others, with the goal of reducing stress, creating job opportunities, and enhancing women’s abilities and literacy. The union currently has over 300 registered members, most of whom have full-time or part-time experience in online microbusiness. To ensure a diverse sample, snowball sampling and convenience sampling were used to select participants. The survey conducted for this study was exempted from IRB review by the IRB Board of SolBridge International School of Business. The decision was based on the fact that the study posed no harm to the respondents, except for possible fatigue from completing the survey, and no personal identifiable data were collected. Additionally, consent was obtained from the respondents in written form at the beginning of the survey, ensuring their voluntary participation.

In May 2022, the survey was administered through an online survey platform (www.wjx.com), and 189 questionnaires were returned. After carefully scrutinizing the responses and eliminating any unreasonable answers, 160 valid questionnaires were selected, accounting for 84.7 percent of the total. The structural equation model (SEM) was used for analysis using AMOS software version 23. The data is available in S1 Dataset.

### Measurement

The questionnaire used in this study had three sections. The first section focused on Entrepreneurial Motivation and utilized a 16-item scale taken from the Entrepreneurial Profile Questionnaire (EPQ) [66,67]. Responses were collected on a five-point Likert-type scale, ranging from "strongly disagree" to "strongly agree".

The second section was divided into two parts: financial performance and non-financial performance. It consisted of a total of ten items on the scale. Financial performance included perceived performance compared to competitors, such as return on assets, financial liquidity, and net profits, while non-financial measures included goal achievement, job creation, performance satisfaction, social contribution, work efficiency, and customer satisfaction. Since online micro-enterprises’ financial performance is not required to be made public, and owners may be hesitant to reveal actual financial figures, a Likert scale with five possible outcomes, ranging from "extremely low performance" to "extremely high performance," was utilized to assess perceived venture performance more accurately. This scale was developed based on business owners’ perceptions of how their company fared against its biggest rival in the market.

The third section of the questionnaire contained 12 different items to gather business profile (industry sector, products, or forms currently in operation) and demographic (gender, age, education level, marital status, childbearing status, role of breadwinner, previous work experience, and managerial position) data.

## Results

### Demographic profile

Based on the survey results presented in Table 1, the female microbusiness entrepreneurs surveyed are primarily aged between 25–54 years old (90.1 percent). In terms of education, the majority of respondents had completed some college education (45.6 percent). Most of the respondents were married (60.6 percent), with almost 40 percent (39.4 percent) having only one child. Within the family, the majority of respondents played a secondary breadwinner role (67.5 percent). Almost all respondents had prior work experience before starting their own business (96.9 percent), with their working years mainly concentrated in the 3–10 year range (70 percent). More than three-fifths (61.3 percent) of respondents had previously held a managerial position. Finally, there was no dominant product or service offered by the respondents.

**Table 1:** Demographic profiles distribution.

Demographic	Value	Frequency	%
Age	18–24	8	5
	25–34	55	34.4
	35–44	51	31.9
	45–54	39	23.8
	55–65	7	4.4
	Over 65	1	0.6
Education	High school degree and below	11	6.9
	Some college	73	45.6
	Bachelor’s degree	56	34.4
	Master’s degree and above	21	13.1
Marital status	Married	97	60.6
	Divorce-separated	34	21.3
	Never married	29	18.1
Childbearing status	No children	36	22.5
	Have a child	63	39.4
	Have two children	49	30.6
	Have three or more children	12	7.5
Role of breadwinner	The only breadwinner	36	22.5
	Secondary breadwinner	108	67.5
	No breadwinner responsibilities (or stress)	16	10
Previous work experience	None	5	3.1
	1–2 years	14	8.8
	3–5 years	61	38.1
	6–10 years	51	31.9
	10–20 years	26	16.3
	More than 20 years	3	1.9
Ever held managerial position	Yes	98	61.3
	No	64	38.7
Products or forms currently in operation	Maternal and baby products	20	12.5
	Skin care cosmetics	33	20.6
	Agricultural and sideline food	23	14.4
	Clothing shoes bags	27	16.9
	Health products	27	16.9
	Platform agency retailer	29	18.1
	Other products	1	0.6

### Descriptive statistics

The descriptive statistics presented in Table 2 reveal that the female microbusiness entrepreneurs in this study exhibit a slightly higher preference for Entrepreneurial Motivation Push (M = 4.27, SD = 0.56) compared to Entrepreneurial Motivation Pull (M = 4.25, SD = 0.47). Furthermore, both financial (M = 3.64, SD = 0.60) and non-financial (M = 3.74, SD = 0.57) performance of the businesses are perceived to be below average. These findings suggest that respondents strongly believe that their motivation levels have an impact on their performance in the workplace.

**Table 2:** Descriptive statistics.

Variables	Obs.	Mean	Std. Dev.	Min	Max
Entrepreneurial Motivation Push	160	4.27	0.56	2.33	5.00
Entrepreneurial Motivation Pull	160	4.25	0.47	2.83	5.00
Business Financial Performance	160	3.64	0.60	2.00	4.75
Business Non-Financial Performance	160	3.74	0.57	2.25	5.00

### Reliability test

The concept of reliably and consistently measuring a phenomenon is known as a measure’s reliability, which plays a crucial role in assessing its effectiveness [68]. In this study, the consistency reliability of the 17 constructs was evaluated using the Cronbach Alpha analysis. The results indicate that all Cronbach’s Alpha values exceed 0.730, indicating high internal consistency. Moreover, the aggregate Cronbach’s Alpha value for all 17 constructs was found to be 0.760, which is significantly higher than the recommended threshold of 0.7 suggested by Cavana et al. [68], indicating strong reliability of the measures used in this study.

### Validity test

Construct validity was evaluated using factor analysis, which was chosen as the preferred method of measurement [68]. The results of the factor analysis are presented in Table 3. The Kaiser-Meyer-Olkin (KMO) score was 0.781, indicating acceptable sampling adequacy. The Bartlett test of sphericity was also statistically significant (p = 0.000; df. = 136), confirming that factor analysis was appropriate for the data. These findings suggest that the items were distinct from one another and supported their respective constructs. Furthermore, based on the results of the component analysis, certain items with lower coefficients were eliminated.

**Table 3:** KMO and Bartlett’s test results.

Kaiser-Meyer-Olkin Measure of Sampling Adequacy.		0.781
Bartlett’s Test of Sphericity	Approx. Chi-Square	648.394
	Df	136
	Sig.	.000

### Measurement model

To evaluate the issue at hand, structural equation modeling (SEM) using AMOS software was employed. The primary objective was to establish the validity of the female entrepreneurial motivation survey and its impact on business performance, as outlined in the hypothesized model. The correlation matrix is presented in Table 4. The validity of the model was evaluated by comparing the composite reliability (CR) and average variance extracted (AVE) values against pre-determined thresholds and test parameters (as presented in Table 5).

**Table 4:** Correlation matrix.

	1	2	3	4
1.Entrepreneurial Motivation Push	1			
2. Entrepreneurial Motivation Pull	0.436**	1		
3. Business Financial Performance	0.133	-0.021	1	
4. Business Non-Financial Performance	0.083	0.193*	0.378**	1

* p < .05

**p < .01.

**Table 5:** Variable validity and reliability.

Variables	Items	Loadings	CR	AVE
Entrepreneurial Motivation Push (MPush)	Desire to give myself, my husband, and children security	0.47	0.619	0.359
	Desire to have greater flexibility in my personal and family life	0.72		
	I met glass ceiling in my work.	0.58		
Entrepreneurial Motivation Pull (MPull)	Desire to keep learning	0.46	0.765	0.354
	Desire to take advantage of an opportunity that appeared	0.58		
	Desire to achieve something and get recognition for it	0.65		
	Desire to make better use of my training and skills	0.61		
	Desire to have freedom to adopt my own approach to work	0.59		
	Desire to be challenged by the startup and growth of a business	0.66		
Business Financial Performance (BFP)	The company’s return on assets (ROA)	0.59	0.709	0.379
	The company’s financial liquidity	0.58		
	The company’s net profits	0.62		
	The company’s sales growth	0.67		
Business Non-Financial Performance (BNFP)	Achieve start up goals	0.67	0.764	0.450
	Satisfaction with company performance	0.76		
	Contribute to the society	0.67		
	Work is efficient	0.57		

According to the literature, AVE values should ideally exceed 0.5, but values above 0.35 are also acceptable [69]. Additionally, if the AVE is less than 0.5, but the composite reliability is higher than 0.6, the construct’s convergent validity is still considered adequate. In this study, all the constructs met the pre-determined thresholds for internal consistency and reliability, suggesting that the model is reliable and acceptable. Therefore, it can be concluded that both the group response and sample are reliable, and the findings can be deemed valid.

### Structural model assessment

**Fig 2:**
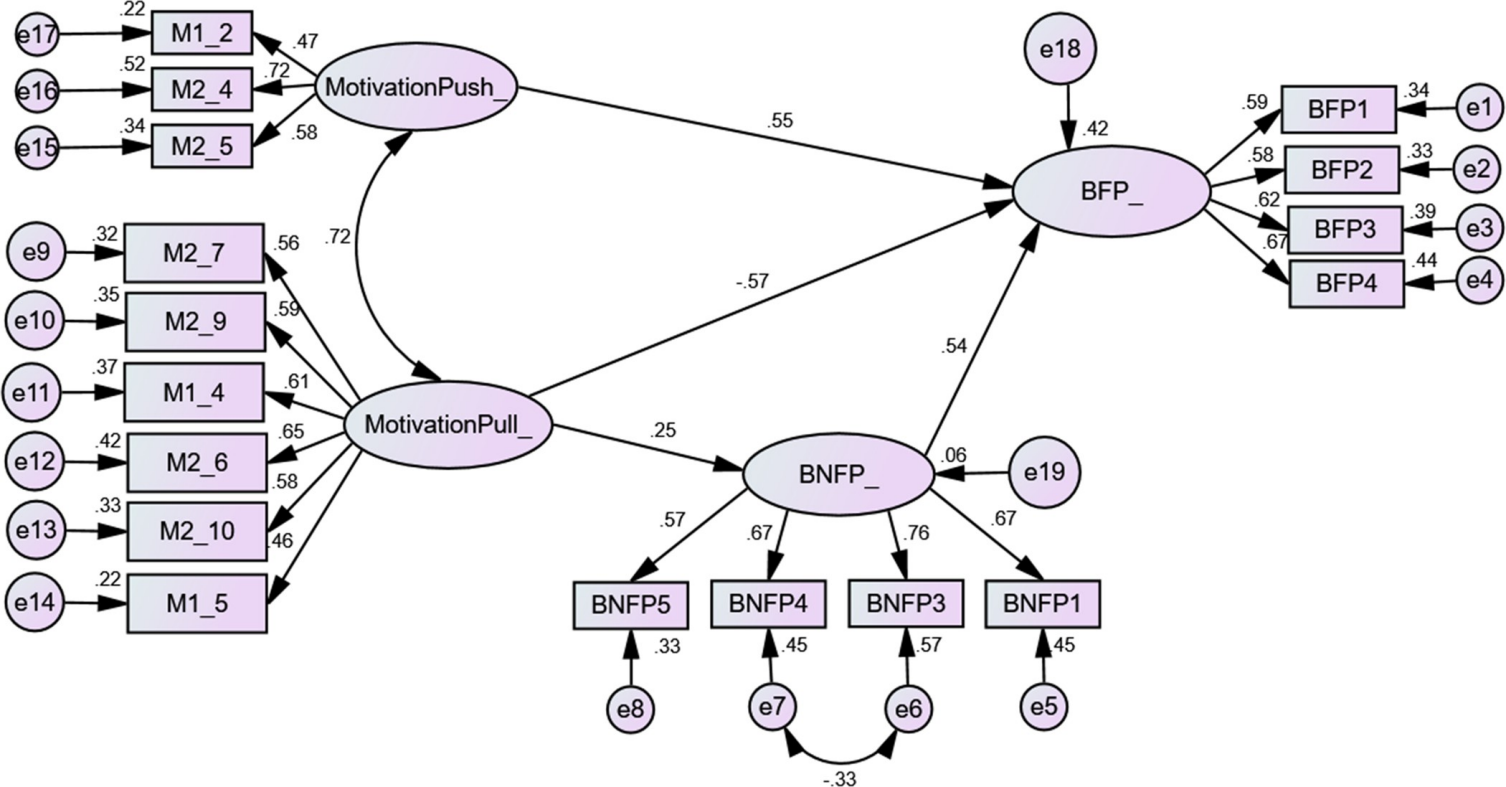
Structural model.

The structural model is presented in Fig 2. As indicated by the figure, some connections beyond the hypotheses were established for the sake of model fit.

Table 6 displays the results of the model fit analysis, indicating that the GFI (0.914) and CFI (0.971) values both exceed the recommended threshold of 0.9, suggesting a good model fit. Additionally, the SRMR value, which is a measure of the difference between the observed and predicted covariance matrices, was 0.035, indicating a satisfactory model fit as it is below the acceptable threshold of 0.10 [70]. Moreover, the RMSEA value (0.029) was lower than the recommended threshold of 0.08, and the CMIN/DF value (1.137) did not exceed the required value of 3, indicating a reasonable fit of the model. Overall, the model’s goodness of fit is considered satisfactory.

**Table 6:** Model fit indices.

SRMR	GFI	CFI	RMSEA	CMIN/DF
0.035	0.914	0.971	0.029	1.137

### Hypothesis testing results

Furthermore, to evaluate the importance of the associations identified in the study, the hypotheses were tested for goodness of fit. The results of the hypothesis testing are presented in Table 7.

**Table 7:** Final results on hypotheses.

Hypothesized Relationships	Standardized Estimates	t-value	P-value	Outcome
H1: MPush -> BFP	0.552	2.067	0.039	Supported
H2: MPull -> BNFP	0.248	2.287	0.022	Supported
H3a: BNFP -> BFP	0.528	4.147	0.000	Supported
H3b: MPull ->BNFP -> BFP	0.180	-	0.040 (0.011,0.514)	Supported
H4: MPull -> BFP	-0.567	-2.224	0.026	Not Supported

The first hypothesis (H1) assesses whether MPush has a substantial impact on the financial performance of the business (BFP). According to the findings, MPush significantly affects BFP (ß = 0.552, t = 2.067, p = 0.039 < 0.05). Thus, H1 is supported.

The second hypothesis (H2) investigates whether entrepreneurial motivation pull (MPull) significantly influences the development of business non-financial performance (BNFP). The findings revealed that MPull has a significant effect on BNFP (ß = 0.248, t = 2.287, p = 0.022 < 0.05). Therefore, H2 is supported.

The third hypothesis (H3) investigates the connection between pull motivation and business financial performance, taking into account the mediating role of business non-financial performance (BNFP). The findings reveal a significant positive effect (ß = 0.528, t = 4.147, p = 0.000 < 0.05) between the financial performance of the business (BFP) and business non-financial performance (BNFP). Additionally, there is a significant negative effect between pull motivation and financial performance (ß = -0.567, t = -2.224, p = 0.026 < 0.05) and a significant indirect effect for the mediation of non-financial performance on the relationship between pull motivation and financial performance (ß = 0.180, p = 0.040 < 0.05, 95% confidence interval does not include 0). Consequently, both H3a and H3b are supported. Moreover, the mediation is partial in nature as the direct effect of pull motivation on business financial performance is significant but negative [71].

The fourth hypothesis (H4) explores whether entrepreneurial motivation pull (MPull) has a significant influence on business financial performance (BFP). The results indicate that MPull does indeed have a significant effect on BFP (ß = -0.567, t = -2.224, p = 0.026 < 0.05). However, contrary to predictions, the impact of MPull on BFP is predominantly negative rather than positive. This unexpected finding suggests that the unique characteristics of female microbusinesses might play a role, warranting further investigation. As a result, H4 is not supported.

### Cross-group analysis

To further explore the characteristics and differences of Chinese female microbusiness entrepreneurs, this study conducted a cross-group analysis of demographic information for opportunity-based and necessity-driven female entrepreneurs. Based on the survey results, respondents whose average score of Entrepreneurial Motivation Push was higher than their average score of Entrepreneurial Motivation Pull were categorized as necessity-driven female entrepreneurs, while those whose average score of push motivation was lower than their average score of pull motivation were categorized as opportunity-based female entrepreneurs. Respondents whose Entrepreneurial Motivation Push and Entrepreneurial Motivation Pull scores were the same were excluded from the analysis. A total of 63 people in the necessity-driven female entrepreneur group and 79 people in the opportunity-based female entrepreneur group were included in the analysis. The results of the cross-group analysis are presented in Table 8.

**Table 8:** Cross-group analysis of demographic information.

Demographic	Value	Opportunity-based female entrepreneurs		Necessity-driven female entrepreneurs	
		Frequency	%	Frequency	%
Age	18–24	3	4.8	5	6.3
	25–34	27	42.9	24	30.4
	35–44	21	33.3	23	29.1
	45–54	10	15.9	22	27.8
	55–65	2	3.2	4	5.1
	Over 65	0	0	1	1.3
Education	High school degree and below	2	3.2	5	6.3
	Some college	31	49.2	33	41.8
	Bachelor’s degree	22	34.9	29	36.9
	Master’s degree and above	8	12.7	12	15.2
Marital status	Married	39	61.9	48	60.8
	Divorce-separated	8	12.7	19	24.1
	Never married	16	25.4	12	15.2
Childbearing status	No children	19	30.2	16	20.3
	Have a child	26	41.3	32	40.5
	Have two children	17	27.9	23	29.1
	Have three or more children	1	1.6	8	10.1
Role of breadwinner	The only breadwinner	11	17.5	19	24.1
	Secondary breadwinner	47	74.6	49	62
	No breadwinner responsibilities (or stress)	5	7.9	11	13.9
Previous work experience	None	1	1.6	4	5.1
	1–2 years	9	14.3	5	6.3
	3–5 years	29	46	26	32.9
	6–10 years	19	30.2	27	34.2
	10–20 years	5	7.9	14	17.7
	More than 20 years	0	0	3	3.8
Ever held managerial position	Yes	34	54	52	65.8
	No	29	46	27	34.2
Products or forms currently in operation	Maternal and baby products	11	17.5	10	12.7
	Skin care cosmetics	14	22.2	18	22.8
	Agricultural and sideline food	3	4.8	3	3.8
	Clothing shoes bags	4	6.3	8	10.1
	Health products	7	11.1	14	17.7
	Platform agency retailer	15	23.8	14	17.7
	Other products	9	14.3	12	15.2

After analyzing the data, we observed that the proportion of opportunity-driven female entrepreneurs under the age of 35 was marginally higher than that of necessity-driven female entrepreneurs. The average age of necessity-driven female entrepreneurs was also found to be slightly older compared to their opportunity-driven counterparts. One plausible explanation for this finding could be that most women have already completed the processes of marriage and childbearing by the age of 35 and are now juggling the responsibilities of motherhood and wifehood, which often leads to greater financial stress. Therefore, older female entrepreneurs might experience a stronger push incentive to establish a business.

Both groups had similar demographic structures. In terms of marital status, the proportion of divorced or separated necessity-driven female entrepreneurs was higher than the proportion of unmarried opportunity-driven female entrepreneurs. One plausible explanation is that unmarried women might still be living with their parents; thus, their cost of living would be lower than that of separated or divorced women. Therefore, divorced or separated women are more likely to be influenced by push factors, whereas unmarried women are more likely to start a business due to pull factors.

Regarding childbearing status, the proportion of female entrepreneurs who have no children is higher among opportunity-driven female entrepreneurs, while necessity-driven female entrepreneurs have more than three children. Thus, female entrepreneurs who are more concerned with raising children are more vulnerable to push factors.

In terms of previous work experience, necessity-driven female entrepreneurs work more hours than opportunity-driven female entrepreneurs. This could be because professional women with more work experience are more susceptible to job satisfaction and the glass ceiling effect.

Regarding the products or forms currently in operation, opportunity-driven female entrepreneurs are more interested in products favored by women, such as clothing, shoes, bags, and health products. Meanwhile, necessity-driven female entrepreneurs have a higher preference for platform-based sales with lower operating costs and investment compared to opportunity-driven female entrepreneurs. The summary of the characteristics of the two groups is presented in Table 9.

**Table 9:** Characteristics of different groups.

	Opportunity-based female entrepreneurs	Necessity driven female entrepreneurs
Age	High proportion of women under 35 years old	Low proportion of women under 35 years old
Marital status	High proportion of unmarried women	High proportion of divorced or separated women
Childbearing status	High proportion of women with no children	High proportion of women with more than 3 children
Previous work experience	Less	More
Products or forms currently in operation	Prefer the project with lower operating cost and investment	Prefer Mainstream products

## Discussion

This study provides a comprehensive explanation of the link between entrepreneurial push motivation and pull motivation, and its impact on business performance in the field of female online microbusinesses in China. The study findings show that push motivation has a positive influence on pull motivation and a significantly favorable impact on financial success, whereas pull motivation has a significant favorable effect on non-financial business performance but does not significantly influence financial performance. The study also examines the role of non-financial performance as a mediator in the link between pull motivation and financial performance, with the result showing that non-financial performance plays a crucial role in improving financial performance.

One significant finding of this study is that female entrepreneurs with pull motivation prioritize achieving non-financial performance first, such as market acceptance, customer satisfaction, and stakeholder support. This finding aligns with Buttner and Moore’s research [31], suggesting that women entrepreneurs motivated by pull factors are more inclined towards intrinsic rewards like personal growth and learning new skills, rather than extrinsic rewards such as financial success or profits. Interestingly, although not part of the study’s hypotheses, it was observed that pull motivation had a significant negative effect on financial performance, which indirectly supports this notion.

It also makes logical sense that women starting a business to realize a recognized opportunity would focus on strategizing and convincing others about the existence of that opportunity. This aligns with the predictions of the goal-setting theory [34,36,37], where individuals invest effort and attention in goals they deliberately or unintentionally set. On the other hand, those with push motivation are primarily concerned with financial performance, as their livelihoods depend on the success of their businesses. This notion was confirmed by this study, where push motivation had a positive and significant effect on financial performance.

Once the non-financial goals are achieved, there is a significant improvement in the financial performance of pull driven entrepreneurs, as evident from the substantial mediation effect. This finding aligns with the research conducted by Ittner and Larcker [62], Kaplan and Norton [16], and Pham [63], which also demonstrated the mediating role of non-financial performance in the impact of strategic initiatives on financial performance. Ittner and Larcker [62] found positive relationships between customer satisfaction measures and future accounting performance, such as revenue and market value. Kaplan and Norton [16] reported that companies that developed a balanced scorecard, a strategic map linking non-financial and financial performance, experienced improved bottom lines. Pham [63] analyzed 211 companies in Vietnam, and found that non-financial performance, measured by improved quality, innovativeness, and market share, fully mediated the relationship between TQM practices and financial performance. The finding that non-financial performance serves as a prerequisite for financial performance is also in line with the predictions of the resource-based theory (RBV), which posits that building a competitive advantage, a foundation for financial success, requires acquiring valuable, rare, difficult-to-imitate resources that can be effectively exploited by the organization (VRIO framework) [58,59].

The cross-group analysis reveals that opportunity-based female entrepreneurs tend to be younger, unmarried, with fewer family responsibilities, and they choose microbusiness initiatives with modest operational expenses and investment. On the other hand, female entrepreneurs driven by necessity have greater family burdens and more work experience. Notably, a high level of necessity-driven motivation among female microbusiness entrepreneurs, as indicated by their strong need to survive financially, contributes to achieving high levels of financial performance.

In contrast, a high level of motivation among opportunity-based female microbusiness entrepreneurs, reflected in their strong desire to express personal values and ambitions, leads to a focus on achieving high non-financial performance initially, before eventually translating into high financial performance. This finding aligns with Buttner and Moore’s research [31], which suggests that women entrepreneurs motivated by pull factors are more inclined towards intrinsic rewards like personal growth and learning new skills, rather than extrinsic rewards such as financial success or profits. It also supports the idea from the goal-setting theory that both deliberate and unintentional goals energize individuals to strive hard to achieve their objectives [34,36,37].

Moreover, the study suggests that family-oriented female microbusiness entrepreneurs will continue to play an essential role in the development of China’s socialist market economy. Small, female-entrepreneur-led microenterprises can develop in China, but their evolution into larger companies is constrained by the country’s intricate social context [7]. To help Chinese female microbusinesses establish and grow lawfully and quickly, further research is necessary.

## Conclusion and implications

This study provides theoretical guidance for Chinese female microbusiness entrepreneurs in grasping the relationship between entrepreneurial motivation and business performance. It highlights the importance of push motivation in achieving financial success and the role of non-financial performance in improving financial outcomes. The study also serves as a resource for other researchers and managers interested in understanding the entrepreneurial motivation, psychology, and group characteristics of Chinese female microbusiness entrepreneurs.

The findings of this study hold significant implications for policymakers and stakeholders aiming to promote the growth of female-led microbusinesses in China. The results indicate that pull-driven entrepreneurs, who are typically young and unmarried, consciously decide not to pursue growth or financial performance beyond a certain level. Research by Morris et al. [46] suggests that women are more likely to opt for a high-growth strategy for their business when they have an equity partner and when they employ workers. This implies that policymakers could foster greater ambition among women entrepreneurs by facilitating the establishment of legal entities other than sole proprietorships and implementing less burdensome regulations for employing workers. These measures could create an environment conducive to female entrepreneurs scaling their businesses and pursuing higher levels of growth and financial success.

In conclusion, this study sheds light on the unique motivations and challenges faced by female entrepreneurs in China. The findings provide useful insights into the factors that influence the performance of female-led microbusinesses and highlight the importance of non-financial performance in achieving financial success. The study suggests that further research is necessary to help Chinese female microbusinesses establish and grow lawfully and quickly, and policymakers and stakeholders should pay attention to the specific needs of family-oriented female entrepreneurs. Overall, this study contributes to our understanding of the role of female entrepreneurs in economic development and provides practical guidance for promoting the growth of female-led microbusinesses in China.

## Limitations and future research directions

While the current study provides valuable insights into the link between entrepreneurial motivation and business performance among female microbusiness entrepreneurs in China, there were certain limitations that need to be considered in future research.

Firstly, it is important to note that while the study found that entrepreneurial pull motivation does not have a direct positive impact on financial performance in the near term, it is still necessary for female microbusiness entrepreneurs to focus on non-financial performance in order to promote long-term financial success. However, the impact of time on female entrepreneurial ambition and firm performance is complex and difficult to capture through data and research. Therefore, future studies could include more temporal elements to better understand this relationship.

Moreover, while the current study provided some initial explanations for the negative influence of entrepreneurial pull motivation on financial performance, further rigorous verification is needed to fully understand this phenomenon. Investigating and describing the link between pull factors of entrepreneurial motivation and business financial performance for female microbusinesses would be a valuable area for future research.

Additionally, as the study highlighted, the role of family and work balance cannot be overlooked in the context of female entrepreneurship in China. Therefore, future research could broaden the study by including factors related to family dynamics and their impact on female entrepreneurial motivation and performance.

In conclusion, while the current study provides important insights into the relationship between entrepreneurial motivation and business performance among female microbusiness entrepreneurs in China, there are still some limitations that need to be addressed in future research. By expanding upon the findings of this study and including additional factors, researchers can gain a more comprehensive understanding of the unique challenges and opportunities faced by female microbusiness entrepreneurs in China and develop strategies to support their success in the increasingly competitive marketplace.

## Supporting information

S1 Dataset (XLS)
